# Supplementary material for: Impact Evaluation of Score Classes and Annotation Regions in Deep Learning-Based Dairy Cow Body Condition Prediction
Source: Animals (Basel). 2023 Jan 4;13(2):194. doi: 10.3390/ani13020194 (PMC9855016; doi:10.3390/ani13020194)
Supplement: Supplementary file 1 [file animals-13-00194-s001.zip › animals-2028714-supplementary.pdf]

Supplementary Materials

Automated 3 box size annotation

After the initial annotation, the position and size of the rectangles on the images were standardized as follows. Three rectangles of different sizes were placed on the images of the F3 farm by the expert annotator (Figure 1). The large (l) box in the image of the animal was placed across the entire width of the rump from the tail head to mid-thigh. The medium (m) box contained the entire width of the rump from the tail head to the base of the vulva. While the small (s) box frames the ischial tuberosities and the tail head. We created a training set from 80% of these images and 20% a validation set. These were used to train the R_50_FPN_3x pre-trained model of the Detrectron2 environment model zoo. During the training, we recorded the validation loss and AP values every 100 iterations and kept the weights that gave the best results. Using the trained model, we predicted the three types of annotation squares for all images and used them for all the main experiments. These bounding boxes were validated visually by the expert annotator.

12 class training and testing after re-labeling test sets into T1-4 classes

Within the test set, for both the T1 and T2 target ranges, the kappa and the accuracy are slightly higher for networks taught in 3 classes than for those taught by 12 classes. For T3 and T4, the opposite is seen. The kappa shows the same trend, with the difference that for the target range T4, the s box shows a higher value than the network taught by 3 class. Within the validation set, the accuracy values of the predictions taught by 12 classes were generally higher. The exception is the m box for the T1 and T2 target domains. For all target domains, the s box resulted in higher kappa for the networks taught by 12 classes. In the target ranges T1 and T2, the l box resulted in a higher kappa for networks taught in 3 classes, while it resulted in a lower kappa for the target ranges T3 and T4. The kappa values of the m boxes were higher for CNNs taught by 3 classes in all target ranges.

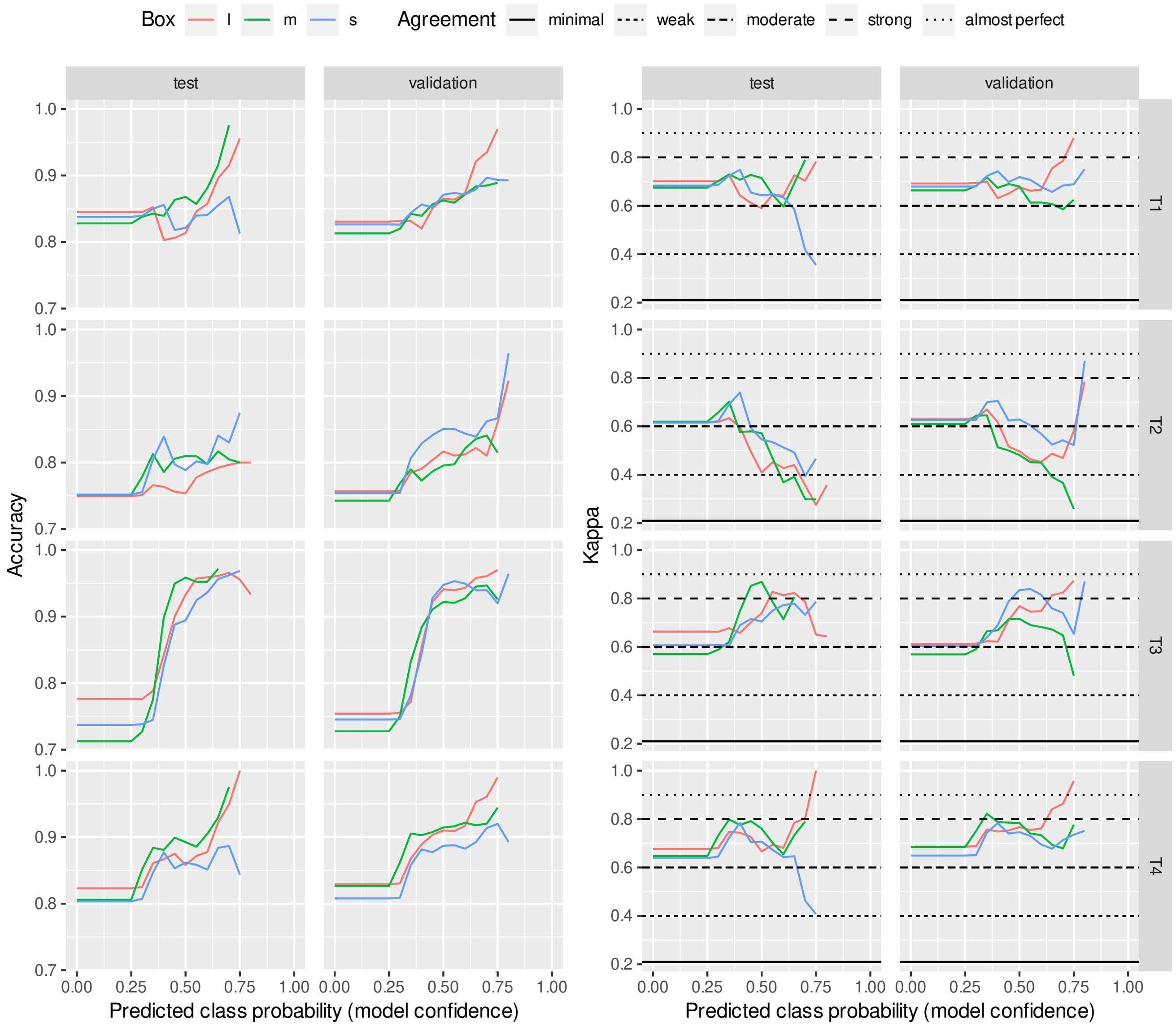

Figure S1: Model trained and evaluated with 12 and 3 BCS classes, respectively. Prediction confidence values (accuracy, Cohen's kappa) are estimated on test and validation sets as a function of predicted class probability. The horizontal lines represent the thresholds to interpret Cohen's kappa values.

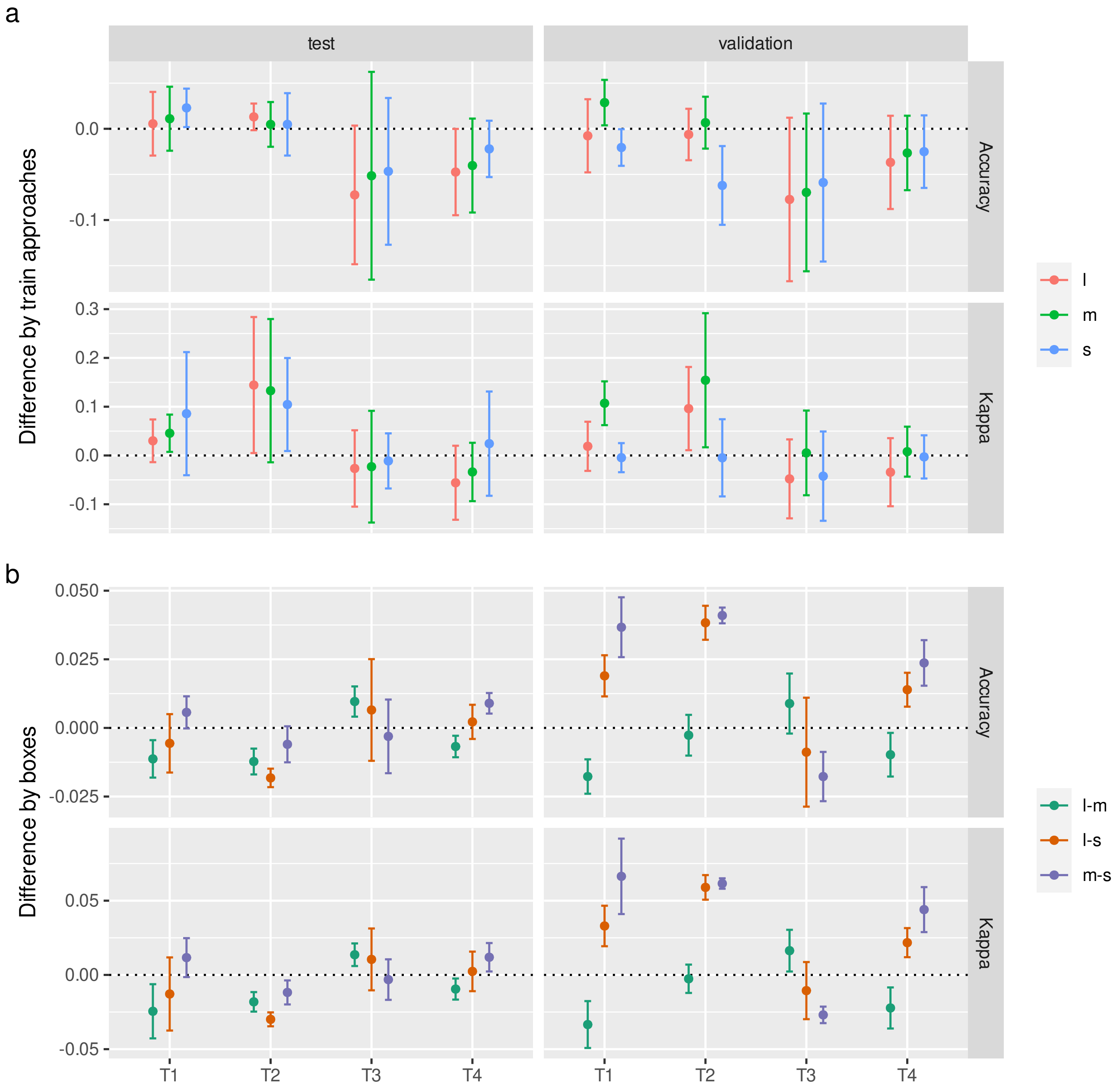

Figure S2: Prediction precision variability shown by mean and standard deviation. Sub-figure a represents the difference between the predictions based on the 3- and 12-class training. Differences among the box sizes in prediction precision obtained from the 3-class train are summarized in sub-figure b.
